# A proteomic perspective on TNF-mediated signalling and cell death

**DOI:** 10.1042/BST20211114

**Published:** 2022-02-15

**Authors:** Maria C. Tanzer

**Affiliations:** Department of Proteomics and Signal Transduction, Max-Planck Institute of Biochemistry, Martinsried 82152, Germany

**Keywords:** apoptosis, necroptosis, post-translational modification, proteomics, tumor necrosis factors

## Abstract

The tumour necrosis factor (TNF) is the most potent inducer of cell death amongst cytokines. It is crucial for processes including homeostasis, the development of the immune system and fighting infections. However, high levels of TNF due to genetic disorders or persistent infections can contribute to autoinflammatory and autoimmune diseases or life-threatening conditions like sepsis. These diseases generally display increased levels of cell death, which, downstream of the TNF receptor, can either be caspase-dependent (apoptosis) or caspase-independent (necroptosis). Significant efforts have been invested in unravelling and manipulating signalling mechanisms regulating these two different types of cell death. Here I discuss how modern proteomic approaches like phosphoproteomics and secretomics provide a novel perspective on this central cytokine and its effect on inflammation and cell survival.

## Introduction

The pro-inflammatory cytokine TNF is a master regulator of the immune system. It is produced by a range of immune cells of the adaptive and innate immune system [1]. TNF enhances antigen-specific responses and is important for the resolution phase of the adaptive immune response [2]. Investigations into TNF expression in innate immune cells revealed its up-regulation during various pathogenic infections. TNF stimulation, in turn, leads to the up-regulation of hundreds of target genes important to fight infections by recruiting other immune cells and amplifying the immune response. Sustained high levels of TNF in persistent infections or autoimmune disorders can have detrimental consequences [3]. This also applies to other proinflammatory cytokines like Interleukin-1β (IL-1b) and Interleukin-6 (IL-6).

However, in contrast with these cytokines, TNF potently induces cell death, which greatly contributes to its pro-inflammatory nature [4]. While cell death of infected cells can prevent further proliferation and spread of the pathogen, persistent cell death can damage the host by exacerbating inflammation. Socrates saying that ‘Death may be the greatest of all human blessings' may not always hold true on the cellular level. Intensive research in the past decades have uncovered a range of different ways a cell can die in response to infections and cytokines with different inflammatory consequences [5]. These various cell death pathways have been developed by the host to fight infection, and over years of co-evolution, they have also been harnessed and manipulated to the pathogen's advantage [6]. TNF has been found to directly induce two types of cell death, which can either be caspase-dependent called apoptosis or caspase-independent called necroptosis [4]. To manipulate TNF signalling for therapeutic purposes, its signalling mechanisms have to be studied in detail. Proteomics offers a global, unbiased and discovery-driven approach that is ideally suited to this purpose. In this perspective, I use TNF signalling as a paradigm case to highlight the power of that technology in (immune) signalling. For illustration I focus on two recent global and extensive studies of our group [7,8].

## TNF signalling and cell death

**Figure 1.**
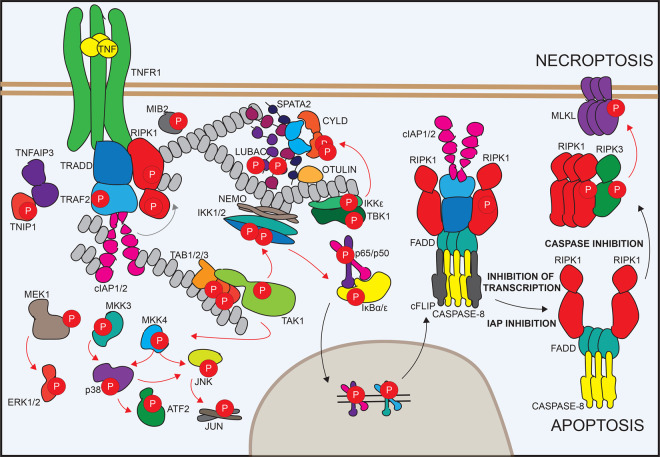
Scheme of TNF signalling including proteins involved in TNF-induced apoptosis and necroptosis. Signalling is primarily mediated through PTMs like phosphorylation and ubiquitylation. Hence, most proteins involved in this signalling are found to be phosphorylated.

The binding of TNF to its TNF receptor induces the recruitment of several cytosolic proteins via mutual domains (Figure 1) [9]. The Fas-associated protein with death domain (FADD), the tumor necrosis factor receptor type 1-associated death domain (TRADD), and receptor-interacting serine/threonine-protein kinase 1 (RIPK1) interact via their death domains with the TNF receptor and each other [10]. Thereby they build a stable signalling platform that allows other proteins to bind, including TNF receptor associated factor 2 (TRAF2) and the E3 ligases inhibitor of apoptosis proteins (IAPs) and the linear ubiquitin assembly complex (LUBAC) complex. Ubiquitination of the complex builds additional recruitment platforms for the kinases transforming growth factor-β-activated kinase 1 (TAK1), IκB kinase (IKKs) and mitogen-activated protein kinase (MAPKs), which upon association can become activated. Intact activation of the nuclear factor ‘kappa-light-chain-enhancer’ of activated B-cells (NF-κB) and MAPK signalling results in the up-regulation of many TNF target genes, including cytokines, chemokines, and also pro-survival proteins such as the cellular FADD-like IL-1β-converting enzyme inhibitory protein (cFLIP), which inhibits caspase activity [11,12]. Therefore, intact TNF signalling does not lead to cell death. Perturbation to this system, including infections, or upon genetic aberrations — which lead to the inhibition, down-regulation, or chronic activation of members of the TNF signalling complex — result in a balance shift from the formation of complex I (pro-survival) to complex II (apoptosis-inducing) with the increased binding and activation of caspase-8/caspase-10 (in human) [3,13,14]. When cFLIP levels are no longer sufficient to keep these caspases in check, they become active and degrade their substrates resulting in apoptosis [15]. Apoptosis is an ‘immunological silent’ cell death, with features including cellular disintegration, blebbing, shrinkage and nuclear fragmentation, with minimal inflammatory stimulation of the environment [4]. Additionally, apoptotic corpses are quickly removed by phagocytes [16]. In several cell types, inhibiting caspases does not block cell death upon TNF stimulation but leads to an alternative, lytic form of cell death called necroptosis [17]. This involves the autophosphorylation of RIPK1, which leads to the activation of the receptor-interacting serine/threonine-protein kinase 3 (RIPK3) and phosphorylation of the mixed-lineage kinase domain-like pseudokinase (MLKL) [18–20]. Active MLKL oligomerises, translocates to the membrane and induces plasma membrane rupture, leading to the uncontrolled release of cellular content, which is thought to drive a strong inflammatory signal [21–26].

## Investigation of TNF signalling using mass spectrometry

**Figure 2.**
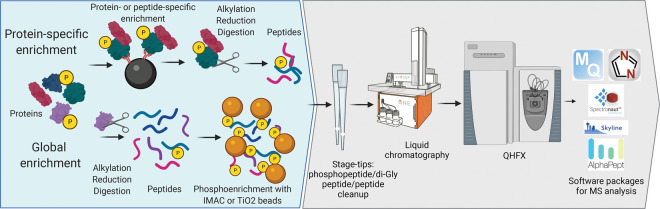
Scheme of proteomic workflows mostly used to investigate signalling changes including post-translational modifications e.g. phosphorylation.

In the last decade, seminal studies investigating TNF signalling used elegant mouse models lacking one or more members of the TNF signalling pathway to analyse their role in homeostasis or upon inflammatory challenges *in vivo* [27–30]. The identification of TNF complex members has primarily occurred through yeast two-hybrid screens and mass spectrometry (MS) [20,31–33]. For example, the central mediator of extrinsic apoptosis caspase-8 was identified in 1996 by Muzio et al. [31] using mass spectrometry, which was then in its infancy. The enormous leap of development and accessibility of this technology enabled the identification and characterisation of many further TNF signalling members [34–37]. Alongside the development of MS technology, genetic screening presented itself as a helpful tool to identify functional members of signalling cascades. MS, however, is the only technology to unbiasedly determine interaction partners in endogenous systems [38]. Just recently several novel members of the TNF signalling complex including the spermatogenesis associated 2 protein (SPATA2), mind bomb 2 (MIB2), the TANK-binding kinase 1 (TBK1) and the IκB kinase epsilon (IKKε) were identified using classical pulldown approaches in combination with MS [34,35,37,39]. MS is also becoming more readily utilised in structural studies and has proven exceptionally successful in combination with various pulldown- and enrichment strategies to unravel enzyme-substrate relationships (Figure 2). It is primarily used in the signalling field to investigate post-translational modifications like phosphorylation, ubiquitylation and less studied PTMs including acetylation, methylation, citrullination and other modifications like protein cleavage [7,35,40]. An enrichment strategy for caspase substrates recently identified the NEDD4-binding protein 1 (N4BP1) as a target of caspase-8 and suppressor of the cytokine response upon lipopolysaccharide (LPS) or TNF stimulation [41]. Also poly(ADP-ribosyl)ation (PARylation) of complex II has been presented as regulator of TNF-induced death, which was discovered through the identification of tankyrase-1 (TNKS1) as a novel interaction partner of complex II via MS (Lin et al. 2021, Biorxiv). Enrichment strategies for these less studied PTMs like PARylation still require optimizations, but offer exciting avenues to unravel novel signalling aspects. MS not only distinguishes between all these different types of modifications, but also reports their precise localisation on the respective substrates and their abundance. A combination of diverse enrichment and/or analysis strategies could provide further insights into the interplay between different PTMs in the same biological context. So far, MS-analyses of TNF signalling was primarily focused on phosphorylation and ubiquitylation due to their pronounced and crucial role. These PTMs were either identified through specific enrichment of the proteins of interest or global phospho- and diGly- enrichments (Figure 2) [7,35,36,42–48]. The subsequent functional analyses and generation of site-specific antibodies validated these findings. A range of studies using MS revealed ubiquitylation of most TNF complex members including the NF-κB essential modulator (NEMO), RIPK1, RIPK3, TRAF2, TRADD primarily mediated by LUBAC and cIAPs, which have also shown to autoubiquitylate [36,49,50]. Further E3 ligases like MIB2, the pellino E3 ubiquitin protein ligase 1 (PELI1) and the TNF-induced protein 3 (TNFAIP3/A20) were also found to regulate TNF signalling, counteracted by DUBs like the OTU deubiquitinase with linear linkage specificity (OTULIN), the cylindromatosis (CYLD) and the ubiquitin specific peptidase 22 (USP22) [51,52]. A study reported that ubiquitylation on four positions of MLKL, which were identified by MS, regulate MLKL-induced cell death upon stimulation and restrain activated MLKL at basal conditions, while another study demonstrated that ubiquitylation of MLKL on a different lysine induces higher-order oligomerisation and activation of MLKL [53,54]. There is a constant development of improved diGly enrichment- and analysis strategies and of new tools that investigate ubiquitin linkages. Additionally, more specific inhibitors targeting E3 ligases and other TNF-signalling members are presented. Together, this will not only provide more information on ubiquitylated proteins and ubiquitin chain types but also offer a starting point for the development of the proteolysis targeting chimera (PROTAC)-based therapies targeting TNF signalling [42,55,56].

## Phosphoproteomics — signalling during cell death

Global PTM enrichment strategies provide a unique, unbiased perspective on the signalling events changing upon specific stimulations. Combined with precise alterations to the system in question, it can provide a snapshot of modified proteins and information about kinetics, enzyme-substrate relationships and localisations of the modified proteins. Recently, we have used this approach on a large scale to elucidate the TNF signalling pathway and signalling events dominating during TNF-induced cell death [7]. A detailed time course experiment revealed different clusters of phosphorylations based on their temporal induction. While we could distinguish early from late events occurring downstream of the TNF signalling receptor, we could not directly discern kinase-substrate relationships due to their rapid on-off kinetics and simultaneous occurrence. *In vitro* kinase assays are frequently used to assign kinases to selected substrates. They are, however, not feasible for large-scale studies [57]. Systematic pulldowns of kinases are also rarely successful in providing a comprehensive and reliable list of substrates due to the mostly transient interactions of kinases with their substrates. For most large-scale approaches like phosphoproteome studies, it is very effective to inhibit the activity or delete various kinases known to orchestrate signalling. While this doesn't prove a direct kinase-substrate relationship, it allows the assignment of regulated phosphorylation events to upstream kinases. Final kinase-substrate relationships of selected candidates can then be determined by *in vitro* kinase assays or by investigating kinase-substrate interactions. Kinase motif analyses can also be of help to pinpoint direct substrates. An earlier study investigated phosphorylation events dependent on RIPK3 by using RIPK3 deficient L929 cells and presented RIPK3 dependent phosphorylations. However, no information on the significance of the phosphorylation changes was reported [45]. Another study presented a deeper phosphoproteome with phosphorylation events downstream of IKK2 using an IKK2 inhibitor upon TNF stimulation [46]. We inhibited a range of kinases driving NF-κB and MAPK signalling, which are known to play a role in TNF signalling and could indeed determine downstream phosphorylation events. Cellular fractionation allowed us to localise these TNF regulated phosphorylation in the nucleus, cytosol and membrane. Combined, this information contributes to a better understanding of the TNF landscape. Another way to determine signalling events important in maintaining intact signalling is to observe regulatory response upon disturbing this balance. One phosphoproteome study investigated TNF signalling upon IAP inhibition, an apoptosis inducing stimulation [44]. This study looked, however, at a very early time point, when caspases are not activated yet. When we triggered TNF-induced apoptosis and necroptosis and looked at phosphorylation changes, we detected evidence of caspase activity specifically during apoptosis based on the activation of the DNA repair pathways. We also observed prominent regulation of transcriptional cyclin-dependent kinases (CDK) phosphorylation upon apoptosis. Zhong et al. [45] showed an increased CDK12 phosphorylation upon TNF treatment in wild-type and RIPK3 knockdown cells, but with no significance reported. With further experiments we revealed a crucial function for CDKs in TNF-induced transcription, affecting levels of target genes like cytokines and pro-survival mediators like cFLIP. Pan-CDK inhibition reduced the induction of all cFLIP isoforms. CDK12/13 inhibition also strongly attenuated up-regulation of cFLIP_L_. Consequently, inhibition of transcriptional CDKs induced synergistic cell death upon TNF stimulation. Hence, our unbiased, global phosphoproteomic approach revealed the organisation of phosphorylations upon TNF stimulation in time and space and highlighted novel potential drug targets that influence the decision between survival and death, inflammation and homeostasis.

## The release of proteins during apoptosis and necroptosis

Our phosphoproteome analysis elucidated cell death type-specific and common signalling events, which can be manipulated to inhibit or enhance cell death. However, information on the inflammatory potential of apoptosis and necroptosis was limited. During apoptosis, the cell membrane integrity remains intact until the later stages of secondary necrosis [58]. Due to the early activation of caspases, many potentially inflammatory components of the cell are cleaved and deactivated, e.g. the damage-associated molecular patterns (DAMPs) like DNA and proteins [59]. *In vivo*, fast removal of apoptotic cells by phagocytes, also known as efferocytosis, prevents the uncontrolled spilling of its content into the environment [16,60]. Therefore, apoptosis is thought to be less inflammatory. In contrast with apoptosis, necroptosis leads to the immediate release of the cellular content and is therefore believed to be highly inflammatory [59,61]. To investigate the inflammatory potential of both modes of cell death in an unbiased way, we analysed the proteins released by apoptotic and necroptotic cells along a time course using mass spectrometry [8]. The supernatants of necroptotic cells showed an early release of a mixture of most intracellular proteins, which supports its presentation as inflammatory cell death. However, when investigating the release of cytokines, we detected a moderate increase in cytokine release by necroptotic cells, in contrast with TNF only treated or apoptotic cells. This is most likely due to the fast kinetics of necroptosis induction *in vitro*. The supernatant analysis also uncovered the activation of specific processes during cell death. We detected extracellular domains of receptors in the supernatants of apoptotic and necroptotic cells. Inhibition of disintegrin and metalloproteinase (ADAMs) demonstrated the activation of these metalloproteases during both cell death types, which leads to the cleavage of surface proteins. This can shut down signalling, detach dying cells from tissue and generate decoy receptors.

Furthermore, we specifically detected an increased release of luminal lysosomal proteins in the supernatant and membrane-bound lysosomal proteins in the extracellular vesicles of necroptotic cells. This indicated the activation of lysosomal exocytosis, a process activated due to membrane perturbation and calcium influx to prevent membrane rupture. Hence, the analysis of supernatants gave indications of the inflammatory potential of cell death types and led to the discovery of processes activated during cell death. In our study, we used a monocytic cell line. Further investigations of primary cells or dying cells in tissue upon more physiological conditions would provide an invaluable addition to the existing data.

## Prospects and challenges

Many studies have already applied various proteomic techniques to successfully investigate different aspects of TNF signalling and TNF-induced cell death. In this way, a range of regulators that present potential therapeutic targets have been identified. In my opinion, we are only at the beginning of exploiting the potential of proteomics in other signalling pathways, especially in primary tissue. So far, the amount of material required for PTM studies has severely limited such exciting projects. However, the increased sensitivity of mass spectrometers and improved protocols that allow efficient sample preparation will mitigate this problem. A perennial challenge in proteomics is identifying key functional events within these large datasets, especially of PTMs, that may contain hundreds to thousands of regulated events. I suggest three approaches could help with this: Firstly, the computational approach, illustrated recently by the development of a method that assigns scores to all phosphorylation events of human proteins reported in Phosphositeplus based on their predicted functionality [62]. The authors considered more than 59 features, including structure, conservation of amino acids and previous experimental results. This yielded a promising overall score that appears to rank the functionally most important phosphorylation events highly. Secondly, the development of an improved stratification of proteins and PTMs by improving the current assignments to enrichment terms and enrichment analysis strategies and by thorough and systematic investigations of enzyme-substrate relationships in diverse biological contexts. While the former can largely be realised with already existing data, the latter requires extensive and well thought out setups involving genetic or pharmacological inhibition in different cell types, with various stimulations at different time points. Thirdly, the combination of proteomic experiments with large, project-specific genetic screens, which help narrow down on proteins and modifications of interest for further follow-up studies.

Besides the incredible technical development on the MS hardware side in the past years, the growing number of software solutions for analysing mass spectrometry data drives the field's expansion, development, and importance. It is highly likely that in the future, proteomics will provide even more insight into signalling pathways and complex biological processes like cell death in diverse biological samples ranging from the single cell to any tissue of interest.

## Perspectives

- Importance of the field. TNF is a prominent and clinically relevant cytokine and a shining example of the potential of proteomic applications to uncover signalling mechanisms. The detailed understanding of signalling pathways and their effects on global cellular processes is a prerequisite for successfully developing therapies.
- A summary of the current thinking. These proteomic approaches can also be applied to unravel other signalling pathways, including other cell death types like pyroptosis, ferroptosis or the process of dead cell removal.
- Future directions. The continuous technical improvement will allow the application of proteomics to investigate various aspects of signalling *in vivo* with spatial information and unprecedented sensitivity.

## Open Access Statement

Open access for this article was enabled by the participation of Max Planck Digital Library in an all-inclusive Read & Publish pilot with Portland Press and the Biochemical Society under a transformative agreement with Max Planck Digital Library.

## Abbreviations

ADAM: A disintegrin and metalloproteinase
CDKs: cyclin-dependent kinases
cFLIP: cellular FADD-like IL-1β-converting enzyme inhibitory protein
CYLD: cylindromatosis
DAMPs: damage-associated molecular pattern
FADD: Fas-associated protein with death domain
IAP: inhibitor of apoptosis protein
IKK: IκB kinase
IKKε: IκB kinase epsilon
IL: interleukin
LPS: lipopolysaccharide
LUBAC: linear ubiquitin assembly complex
MAPK: mitogen-activated protein kinase
MIB2: mind bomb 2
MLKL: mixed-lineage kinase like domain-like
MS: mass spectrometry
N4BP1: NEDD4-binding protein 1
NEMO: NF-κB essential modulator
NF-κB: nuclear factor ‘kappa-light-chain-enhancer’ of activated B-cells
OTULIN: OTU deubiquitinase with linear linkage specificity
PARylation: Poly(ADP-ribosyl)ation
PELI1: Pellino E3 ubiquitin protein ligase 1
PROTAC: proteolysis targeting chimera
PTMs: post-translational modifications
RIPK1: receptor-interacting serine/threonine-protein kinase 1
RIPK3: receptor-interacting serine/threonine-protein kinase 3
SPATA2: spermatogenesis associated 2
TAK1: transforming growth factor-β-activated kinase 1
TBK1: TANK-binding kinase 1
TNF: tumour necrosis factor
TNFAIP3: TNF-induced protein 3
TNKS1: tankyrase-1
TRADD: tumour necrosis factor receptor type 1-associated death domain
TRAF2: TNF receptor associated factor 2
USP22: ubiquitin specific peptidase 22
